# Single-cell RNA sequencing reveals the effects of mental stress on mouse mammary tumors and the tumor microenvironment

**DOI:** 10.1038/s41420-025-02619-1

**Published:** 2025-07-16

**Authors:** Pengfei Liu, Wenjing Ma, Tao Wang, Jinhui Lü, Weizhong Wang, Yixing Wang, Qifan Tang, Jing Di, Evelyne Bischof, Qian Zhao, Zuoren Yu

**Affiliations:** 1 Medical Innovation Center & State Key Laboratory of Cardiovascular Diseases, Shanghai East Hospital, Tongji University School of Medicine, Shanghai, China; 2 Department of Internal Medicine of Traditional Chinese Medicine, Shanghai East Hospital, Tongji University School of Medicine, Shanghai, China; 3 University of Shanghai for Science and Technology, Summer Internship Program, Shanghai, China; 4 Department of Pathology and Laboratory Medicine, University of Kentucky, Lexington, KY USA; 5 Department of Medical Oncology and Clinical Trials Unit, Hospital of the University of Cologne, Cologne, Germany

**Keywords:** Cancer microenvironment, Genetics research

## Abstract

Mental stress has been shown to negatively impact the development and progression of human cancer, including breast cancer. However, its effects on the tumor microenvironment (TME) remain unclear. In this study, we applied single-cell sequencing analysis to tumor tissues from *MMTV-PyMT* transgenic mice with mammary gland tumors with or without exposure to mental stress. In association with a significant promotion of the cell cycle and tumor growth induced by mental stress, we observed the dedifferentiation of luminal subtype of tumor cells into a subgroup of cancer stem cell-like basal cells, as well as enhanced cell proliferation in epithelial tumor cells, endothelial cells, and fibroblasts. In addition, stress stimulation led to an increase in tumor-associated neutrophils (TANs) and tumor-infiltrating dendritic cells (TIDCs), while suppressing immune cells such as cytotoxic T lymphocytes (CTLs), naïve T cells, B cells, and NK cells within the TME. We also observed a shift in macrophages from the M1 to the M2 phenotype. Furthermore, pathway enrichment analysis of differentially expressed genes, gene signature U score analysis, and immunofluorescence staining of the tumor tissue sections were conducted for further validation. The current study not only systematically elucidates the impact of mental stress on mammary gland tumors and the TME in vivo, but also provides insights into the mechanism underlying mental stress-induced tumor growth and progression in breast cancer.

## Introduction

Breast cancer is the most common cancer type and the leading cause of cancer-related death in women worldwide [1]. In addition to the genetic mutations, unhealthy lifestyles and mental factors like depression and anxiety are believed to have a significant influence on the incidence and mortality of breast cancer patients [2–4].

Emerging evidence indicates that mental stress (MS) seriously affects health status and even promotes tumor progression in human cancer [5, 6]. Additionally, stress and anxiety negatively influence the therapeutic outcome and prognosis in patients [7, 8]. Determining the regulatory mechanisms mediating the MS-promoted tumor growth and progression in human cancer including breast cancer is crucial for formulating more effective therapeutic strategies.

The TME is the ecosystem surrounding the tumor that plays a critical role in regulating tumor development, growth, metastasis, and treatment response. The TME is composed of non-tumor cells, extracellular matrix (ECM), blood vessels, and the molecules they produce [9]. Targeting the TME compartments as a novel potential therapeutic approach in the treatment of human cancer has been well discussed and tested, exhibiting a strong potential in clinical application [10, 11]. Preclinical and clinical studies have demonstrated that combination therapies targeting both the TME and tumor cells effectively reduce tumor growth, metastasis, and chemotherapy resistance [12, 13].

The development of the single-cell RNA sequencing (scRNA-seq) technique makes it possible to analyze individual cells within a tumor tissue, explore cellular heterogeneity, identify cell subtypes, and detect cell-cell interactions [14–16]. In this study, scRNA-seq was applied to analyze the TME and tumor cells in a spontaneous breast cancer mouse model with or without exposure to MS stimuli. Promotion of the tumor growth by stress stimulation was confirmed. Alterations in each cell type within the TME were determined, including gene expression, cell subtype, biological function, molecular pathway, and intercellular communication. Our findings provide novel insights for understanding the mechanisms mediating the MS-promoted tumor growth and tumor progression in breast cancer. It holds strong potential for the development of integrated therapeutic strategies targeting both tumor cells and the TME.

## Results

### Impact of stress exposure on mammary tumor growth in mice

**Fig. 1:**
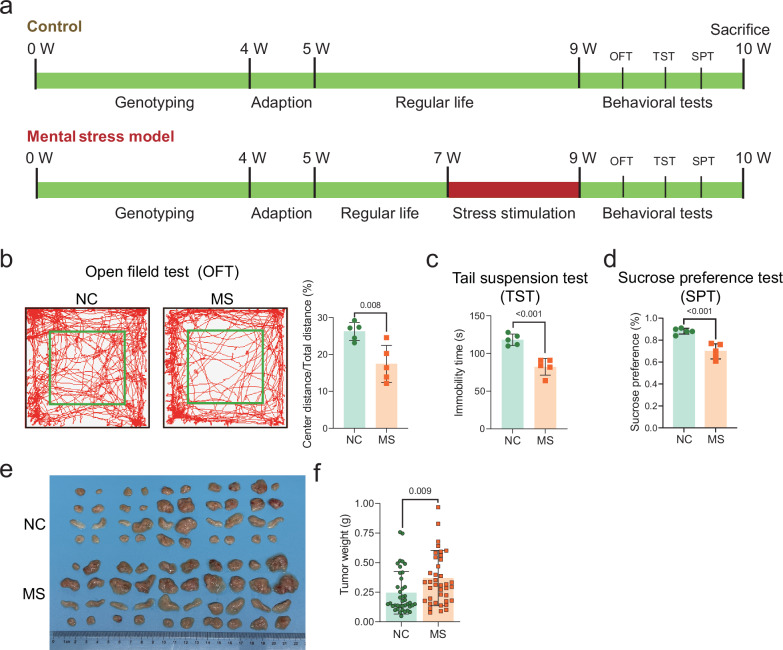
Impact of stress stimuli on the mouse behavior and mammary tumor growth. **a** Schematic presentation of the workflow for *MMTV-PyMT* tumor mice exposure to mental stress stimulation. **b** Mouse behavioral test using the open field test (OFT). **c** Mouse behavioral test using the tail suspension test (TST). **d** Mouse behavioral test using the sucrose preference test (SPT). **e** Images of mammary gland tumors isolated from the MS and NC groups of mice. **f** Tumor weights from both groups of mice. Data are presented as the mean ± SEM (*n* = 5 in **b**–**d**; *n* = 40 in **e**, **f**). MS mental stress, NC negative control.

To confirm the impact of MS on tumorigenesis in breast cancer, multiple stress stimuli were applied to *MMTV-PyMT* female mice from week 8 to week 9 after birth, which corresponds to the appropriate time for this mouse model to generate mammary gland tumors. Three behavioral test assays including the open field test (OPT), the tail suspension test (TST) and the sucrose preference test (SPT) were applied to the mice within one week after finishing all stress stimulations (Fig. 1a). As a result, those mice in the MS group exhibited reduced activity in the center zone in the OPT (Fig. 1b), decreased immobility time in the TST (Fig. 1c), and lower preference for sucrose in the SPT (Fig. 1d), indicating negative effects of stress exposure on mental health in mice. At the age of week 10, all the mice were euthanized. The body weight and the tumor weight were measured. As shown in Supplementary Fig. S1 and Fig. 1e, f, the mice did not show a difference in body weight between the MS and NC groups, whereas the tumor size and tumor weight showed a significant increase in mice of the MS group.

### scRNA-seq analysis of the mammary gland tumors from both MS and NC groups

**Fig. 2:**
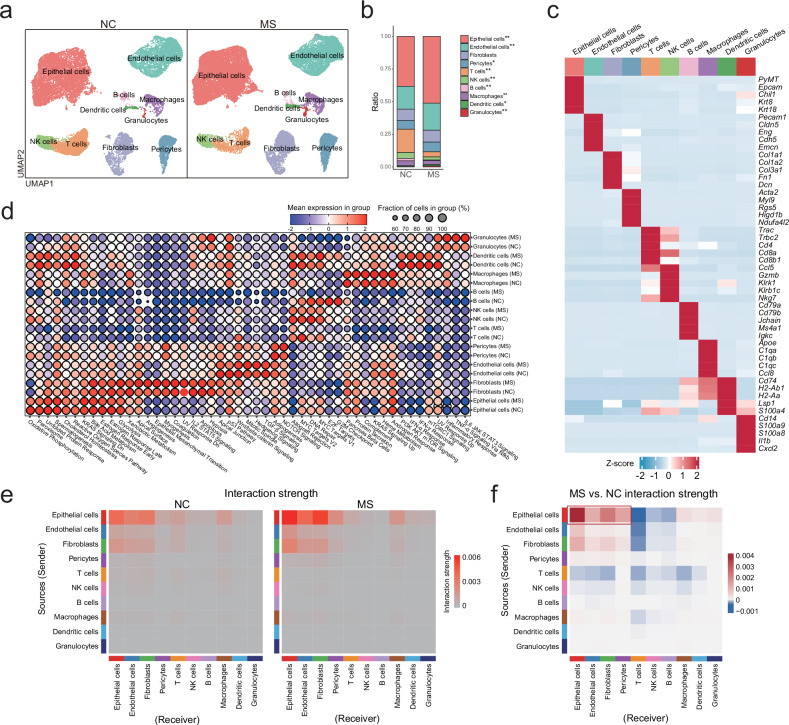
Impact of stress stimuli on the murine mammary TME. **a** UMAP plot of total cells in NC and MS tumors, grouped by cell types. **b** Proportion of each type of cells in total cells. Statistical significance was indicated (Fisher’s exact test, *p < 0.05, **p < 0.01). **c** Heatmap of the average expression levels of the marker genes used for annotation of each cell type. **d** Dot plot displaying the signature enrichment scores of the Hallmark pathway gene sets for each cell type under MS or NC condition. **e** Heatmap showing the strength of cell-cell interactions in MS and NC mice. **f** Heatmap illustrating the differential cell-cell interaction strengths between the MS and NC groups of mice.

Given the critical role of the TME in tumorigenesis, single-cell RNA sequencing was performed on mammary gland tumor tissues from both MS and NC groups of mice. A total of 43,403 cells were detected, including 25,334 from the MS group and 18,069 from the NC group. These cells were classified into 10 major types, including epithelial tumor cells, endothelial cells, fibroblasts, pericytes, T cells, NK cells, B cells, macrophages, dendritic cells (DCs), and granulocytes (Fig. 2a, b). Gene markers used to annotate each cell type are shown in Fig. 2c. As a result, the tumor tissues from the MS group of mice exhibited significant increases in the proportion of epithelial tumor cells, endothelial cells, pericytes, DCs, and granulocytes, while decreases in immune cells including T cells, B cells, NK cells, and macrophages, compared to the NC group of mice (Fig. 2a, b).

Pathway enrichment analysis revealed distinct patterns of the Hallmark pathways in response to stress exposure in different cell types (Fig. 2d). For instance, upon stress stimulation, epithelial tumor cells exhibited elevated activities in cell cycle-related pathways including mitotic spindle, E2F targets, and G2/M checkpoint. Fibroblasts and pericytes showed increased enrichment in pathways of angiogenesis, epithelial-mesenchymal transition, apical surface, and Notch signaling. Notably, p53 signaling showed suppression by stress exposure in epithelial tumor cells, endothelial cells, and fibroblasts as well. Those immune response-related signaling pathways including allograft rejection, IFN-α response, and cell cycle-related pathways including MYC targets, E2F targets, and G2/M checkpoint were suppressed in T cells, B cells and/or NK cells by stress exposure. On the contrary, those inflammatory pathways, such as IL6/JAK/STAT3 signaling, inflammatory response, TNF-α signaling, *etc*., were activated in granulocytes (Fig. 2d).

Additionally, we analyzed the impact of stress exposure on the interactions between different cell types within the TME. As shown in Fig. 2e, f, upon stress stimulation, the interaction strengths between epithelial tumor cells and endothelial cells, pericytes, or fibroblasts were promoted, while the interactions between T cells and epithelial tumor cells, endothelial cells, or fibroblasts were suppressed, which is consistent with the stress-induced tumor growth.

### Stress exposure induced dedifferentiation of luminal subtype of epithelial tumor cells towards cancer stem cell-like basal subtype

**Fig. 3:**
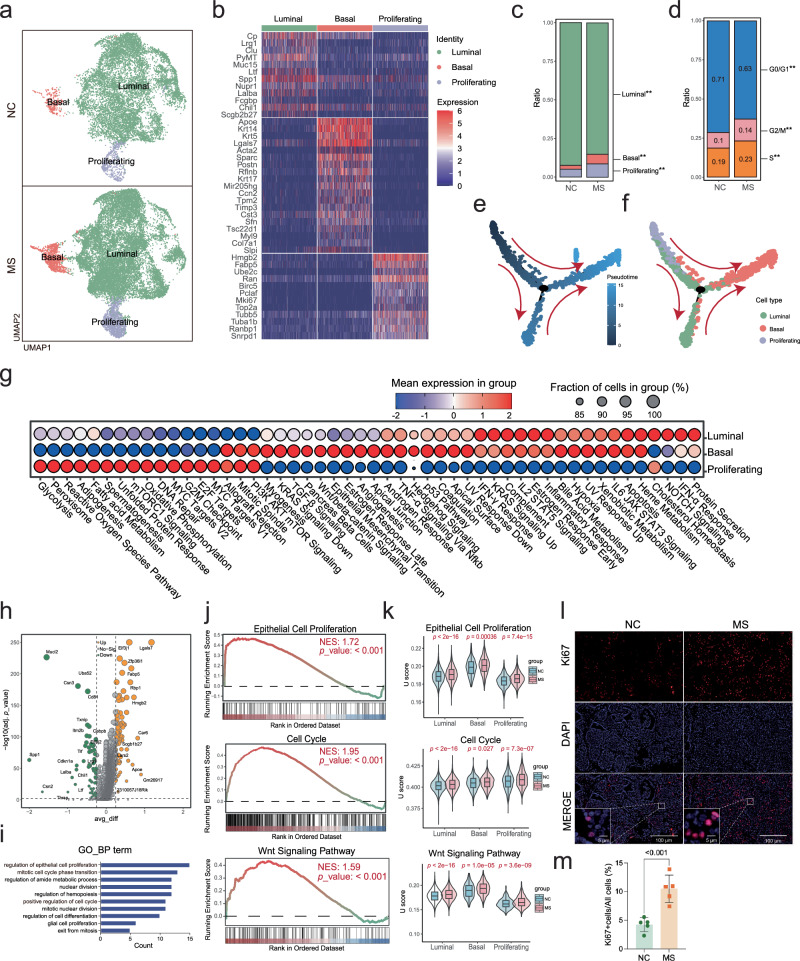
Impact of stress stimuli on epithelial tumor cells within the TME. **a** UMAP plot of epithelial tumor cells in NC and MS tumors, grouped by cell subtypes. **b** Heatmap of the expression levels of the marker genes used for annotation of tumor cell subtypes. **c** Proportion of each epithelial tumor cell subtype in all tumor cells. Statistical significance was indicated (Fisher’s exact test, **p < 0.01). **d** Proportion of tumor cells at different stages of the cell cycle. Statistical significance was indicated (Fisher’s exact test, **p < 0.01). Trajectory analysis of epithelial tumor cells, displayed by pseudotime (**e**) and cell subtype (**f**). Cell differentiation direction was indicated with red arrows. **g** Dot plot of Hallmark pathway signature enrichment score in each subtype of epithelial tumor cells. **h** Volcano plot of DEGs in epithelial tumor cells between MS and NC mice. **i** GO pathway enrichment analysis of DEGs showing the top 10 pathways (adjusted *p* < 0.05). **j** GSEA plot showing representative pathways including epithelial cell proliferation, the cell cycle, and Wnt signaling (MS *vs* NC). **k** The signature enrichment score (U score) of representative pathways, including the epithelial cell proliferation, the cell cycle, and Wnt signaling in epithelial tumor cells (MS *vs* NC). **l** Ki67 immunofluorescence staining analysis of tumor tissue sections. **m** Quantification of the Ki67 staining in **l** (mean ± SEM, *n* = 5).

We categorized the epithelial tumor cells into three subgroups: proliferating (*Mki67*, *Top2a*, *Hmgb2*, *etc*., as marker genes), luminal (*Lrg1*, *Clu*, *Muc15*, *etc*., as marker genes), and basal (*Acta2*, *Krt5*, *Krt14*, *etc*., as marker genes) (Fig. 3a, b), in which the proportions of both proliferating and basal subgroups of tumor cells increased, while the luminal subpopulation decreased in mice exposure to stress stimuli (Fig. 3c). The cell cycle analysis revealed an induction of G0/G1-S transition in tumor cells by stress stimuli (Fig. 3d), which was validated by the expression profiling analysis of *Mki67* in tumor cells (Supplementary Fig. S2a, b). Trajectory analysis indicated a differentiate direction of tumor cells from the proliferating and luminal subgroups to the basal subgroup (Fig. 3e, f, Supplementary Fig. S2c), suggesting a stress-induced tumor cell dedifferentiation. Subsequent hallmark pathway analysis indicated increase of epithelial mesenchymal transition (EMT) and cancer stem cell-regulating pathways, such as Wnt/beta-catenin signaling, KRAS signaling, TGF-β signaling, AKT signaling, etc., in the basal subgroup of tumor cells (Fig. 3g). The proliferating subgroup of tumor cells showed enrichment in the cell cycle- and cell metabolism-related signaling pathways, including E2F/MYC targets, G2/M checkpoint, DNA repair and fatty acid metabolism (Fig. 3g).

DEG analysis in epithelial tumor cells and subsequent pathway analysis demonstrated enrichment in the pathways related to cell proliferation, cell cycle and cell differentiation (Supplementary Table 1, Fig. 3h, i). Gene set enrichment analysis (GSEA) of the DEGs validated their enrichment in pathways regulating cell proliferation and the cell cycle (Fig. 3j). In addition, the signature enrichment score (U score), derived by the AddModuleScore_UCell tool, further confirmed the stress-induced cell proliferation and the cell cycle in epithelial tumor cells (Fig. 3k). Moreover, we applied an immunofluorescence staining analysis of Ki67 to tumor tissue sections, demonstrating a much higher percentage of Ki67 positive cells in the MS tumors, compared to NC (Fig. 3l, m).

### Impact of stress exposure on endothelial cells within the TME

**Fig. 4:**
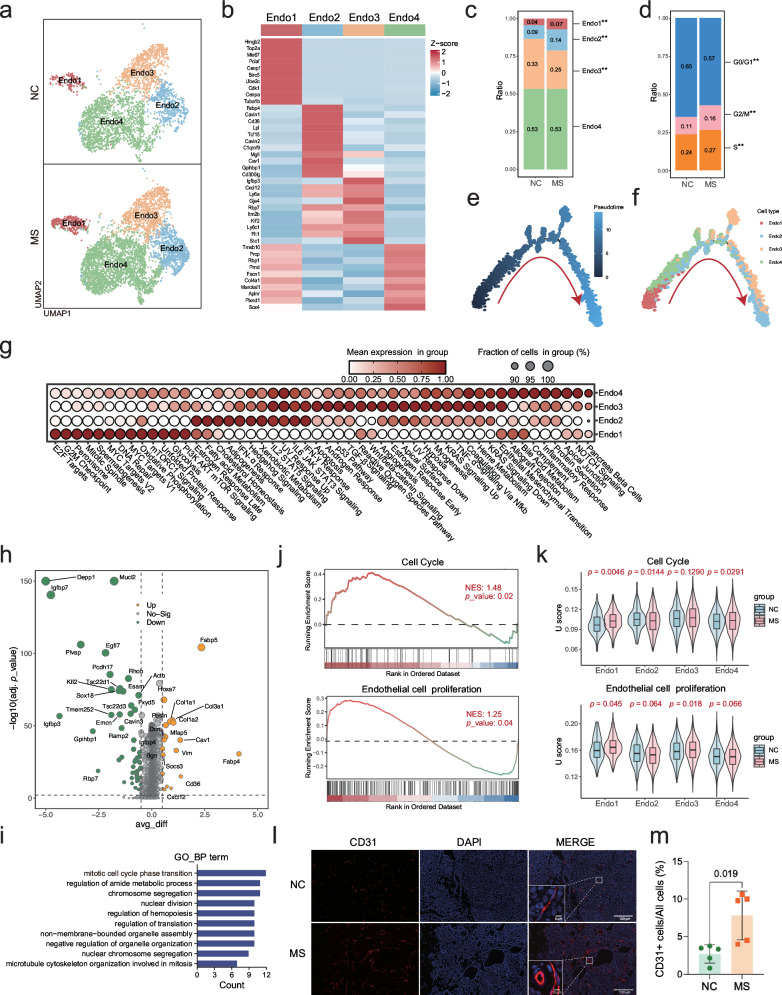
Impact of stress stimuli on endothelial cells within the TME. **a** UMAP plot of endothelial cells in the TME, grouped by cell subtypes. **b** Heatmap of the average expression levels of the marker genes used for annotation of endothelial cell subtypes. **c** Proportion of each endothelial cell subtype in all endothelial cells. Statistical significance was indicated (Fisher’s exact test, **p < 0.01). **d** Proportion of endothelial cells at different stages of the cell cycle. Statistical significance was indicated (Fisher’s exact test, **p< 0.01). Trajectory analysis of endothelial cells, displayed by pseudotime (**e**) and cell subtype (**f**). Cell differentiation direction was indicated with red arrows. **g** Dot plot of Hallmark pathway signature enrichment score in each subtype of endothelial cells. **h** Volcano plot of DEGs in endothelial cells in the TME between MS and NC mice. **i** GO pathway enrichment analysis of DEGs in endothelial cells showing the top 10 pathways (adjusted *p* < 0.05). **j** GSEA plot showing representative pathways including the cell cycle and cell proliferation in endothelial cells (MS *vs* NC). **k** The signature enrichment score (U score) of representative pathways, including the cell cycle and cell proliferation in endothelial cells (MS *vs* NC). **l**. CD31 immunofluorescence staining analysis of tumor tissue sections. **m** Quantification of the CD31 staining in **l** (mean ± SEM, *n* = 5).

As the most important angiogenesis-related cell type in the TME, endothelial cells were classified into four subpopulations in the current study, including Endo1, Endo2, Endo3, and Endo4 (Fig. 4a) by using the marker genes shown in Fig. 4b. Cell quantitative analysis indicated increase of Endo1 and Endo2 subpopulations upon stress exposure (Fig. 4c). The cell cycle analysis revealed a stress-promoted G0/G1-S transition in endothelial cells (Fig. 4d), which was in consistence with the higher level of *Mki67* in endothelial cells of MS tumors (Supplementary Fig. S3a). Uniform Manifold Approximation and Projection (UMAP) plot indicated a high enrichment of *Mki67*-positive endothelial cells in the Endo1 subpopulation (Supplementary Fig. S3b). Trajectory analysis suggested a sequential progression from Endo1/Endo4 to Endo2/Endo3 subpopulations (Fig. 4e, f, Supplementary Fig. S3c). Hallmark pathway analysis clearly indicated that Endo1 subpopulation was enriched with the cell proliferation-related pathways, Endo2 subpopulation with lipid metabolism and adipogenesis-related pathways, Endo3 subgroup with the angiogenesis-related pathways, and Endo4 subpopulation with the angiogenesis- and immune response-related pathways (Fig. 4g). Notably, a comparative analysis of intercellular communications regulating endothelial proliferation and angiogenesis between the stress and control groups was performed using CellChat (Supplementary Fig. S3d–h). The results revealed that pro-angiogenic signaling was significantly enhanced by MS, particularly through the upregulation of VEGF, ANGPT, and ANGPTL pathways between pericytes/fibroblasts and endothelial cells. Additionally, we observed a marked increase in TGF-β and VISFATIN-mediated crosstalk between epithelial and endothelial cells under stress condition. These findings further reinforce the observation of stress-induced cell proliferation and angiogenesis in endothelial cells.

DEGs analysis in endothelial cells between the MS and NC groups found a subset of upregulated genes (*Fabp4*, *Fabp5*, *CD36*, *Cav1*, etc.) and a subset of downregulated genes (*Igfbp3*, *Klf2*, *Igfbp7*, *Depp1*, etc.) upon stress exposure (Supplementary Table 2, Fig. 4h). Consistent with the observations above, GO pathway analysis (Fig. 4i), the GSEA analysis (Fig. 4j), and the signature enrichment score (U score) analysis (Fig. 4k) of endothelial cells validated a significant induction of the cell cycle and endothelial cell proliferation by stress stimulation. Additionally, immunofluorescence analysis of CD31, a marker for vascular endothelial cells, was applied to tumor tissue sections (Fig. 4l), further confirming the phenotypes (Fig. 4m).

### Impact of stress exposure on cancer-associated fibroblasts within the TME

**Fig. 5:**
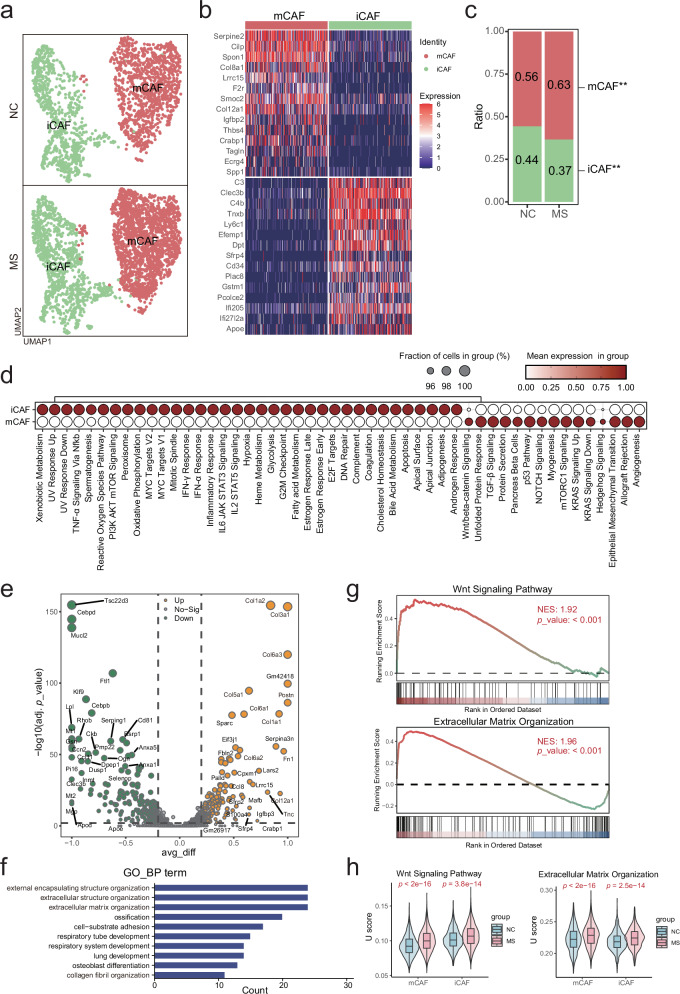
Impact of stress stimuli on fibroblasts within the TME. **a** UMAP plot of fibroblasts in the TME, grouped by cell subtypes. **b** Heatmap of the expression levels of the marker genes used for annotation of two subtypes of fibroblasts. **c** Proportion of each subtype of fibroblasts in all fibroblasts. Statistical significance was indicated (Fisher’s exact test, **p < 0.01). **d** Dot plot of Hallmark pathway signature enrichment score in each subtype of fibroblasts. **e** Volcano plot of DEGs in fibroblasts in the TME between MS and NC mice. **f** GO pathway enrichment analysis of DEGs in fibroblasts showing the top 10 pathways (adjusted *p* < 0.05). **g** GSEA plot showing representative pathways including the extracellular matrix organization and Wnt signaling in fibroblasts (MS *vs* NC). **h** The signature enrichment score (U score) of representative pathways including the extracellular matrix organization and Wnt signaling in fibroblasts (MS *vs* NC).

Fibroblasts, as a predominant cell type within the TME, not only produce large amounts of ECM, but also secrete various cytokines that drive tumor proliferation, angiogenesis, and immune evasion. Cancer-associated fibroblasts (CAFs) exhibit pro-tumorigenic activities to support tumor cell proliferation and invasion [17, 18]. Herein, we clustered CAFs into two subpopulations as myofibroblastic CAFs (mCAFs) (*Col8a1*, *Serpine2*, *Cilp*, *F2r*, *Postn*, *etc*., as marker genes) and inflammatory CAFs (iCAFs) (*C3*, *Clec3b*, *C4b*, *Cd34*, *Cxcl2*, *etc*., as marker genes) (Fig. 5a, b, Supplementary Fig. S4a, b). The proportion of mCAFs showed a little higher than iCAFs in our cases (Fig. 5c). Hallmark pathway analysis indicated that mCAFs were enriched with the angiogenesis-regulating pathways, such as angiogenesis, EMT, TGF-β signaling, and cell stemness-regulating pathways including Notch signaling and Wnt/β-catenin signaling (Fig. 5d). iCAFs were enriched with inflammation response-related pathways and cell cycle-related pathways (Fig. 5d). mCAFs within the TME were induced by stress exposure (Fig. 5c).

DEG analysis in CAFs (Supplementary Table 3, Fig. 5e) and subsequent pathway analysis highlighted their function in regulating ECM (Fig. 5f), which was further validated by the GSEA analysis (Fig. 5g) and the gene signature enrichment U score analysis (Fig. 5h).

According to the literature [19], iCAFs predominantly secrete inflammatory cytokines, including IL-6, IL-8, and CXCL12, to maintain an immunosuppressive and pro-angiogenic niche. In contrast, mCAFs exhibit upregulation of α-SMA and collagen, promoting ECM stiffening and physical barrier formation. The stress-induced transition of iCAFs to mCAFs may represent a shift in the TME from an inflammatory to an invasive condition, which could be at least partly responsible for tumor metastasis, therapy resistance, and immune escape [20].

### Impact of stress exposure on pericytes within the TME

Pericytes regulate angiogenesis and contribute to tumor vascularization in the TME [21]. Our scRNA-seq analysis defined a MS-induced subpopulation of pericytes, named Pericyte1 (Supplementary Fig. S5a), which were distinguished from other pericytes by a set of marker genes including *Mki67*, *Top2a*, *Cenpf*, *Pclaf*, *Hmgb2*, etc. (Supplementary Fig. S5b). Additional analysis indicated that Pericyte1 subpopulation may be produced by the MS-promoted cell proliferation (Supplementary Fig. S5c–f), or by a cellular trajectory differentiation (Supplementary Fig. S5g–i). Hallmark pathway analysis of the Pericyte1 subpopulation demonstrated gene enrichments in the cell cycle, EMT, and angiogenesis (Supplementary Fig. S5j).

DEGs analysis in pericytes (Supplementary Table 4, Supplementary Fig. S5k) and subsequent pathway analysis highlighted their function in regulating ECM and cell-matrix adhesion (Supplementary Fig. S5l). GSEA enrichment analysis of DEGs in Pericyte1 indicated the impact on cell proliferation, ECM assembly and vascular-associated smooth muscle cell migration in pericyte by MS (Supplementary Fig. S5m, n).

### Impacts of stress exposure on T, B, and NK lymphocytes within the TME

T cells, B cells, and natural killer (NK) cells are the main components of the immune system. Cytotoxic T lymphocytes (CTLs) expressing cell-surface CD8 are able to target and destroy cancer cells, and secret cytokines to restrict tumor growth and metastasis, while regulatory T cells (Tregs) suppress CD8^+^ T cells, facilitating tumor immune evasion [22]. Naïve T cells and γδ T cells regulate immune response and immune surveillance [23]. B cells participate in anti-tumor immunity through antibody production, antigen presentation, and cytokine secretion [24]. NK cells are able to kill tumor cells directly and mediate antibody-dependent cellular cytotoxicity [25].

**Fig. 6:**
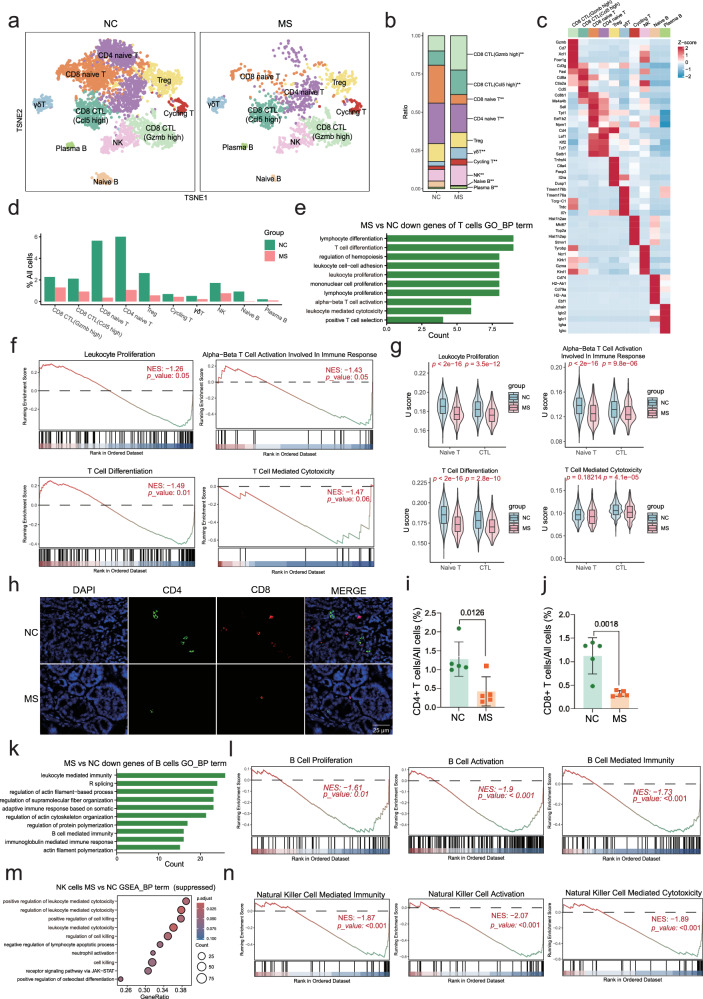
Impact of stress stimuli on T cells, B cells and NK cells within the TME. **a** UMAP plot of T cells, B cells and NK cells in the TME, grouped by cell subtypes. **b** Proportion of each subtype of T, B, and NK cells. Statistical significance was indicated (Fisher’s exact test, **p *<* 0.01)*.* **c** Heatmap of the average expression levels of the marker genes used for annotation of each subtype of T, B, and NK cells. **d** Proportion of each subtype of T, B, and NK cells in all cells. **e** GO pathway enrichment analysis of DEGs in T cells showing the top 10 pathways (adjusted *p* < 0.05). **f** GSEA plot showing representative pathways including leukocyte proliferation, T cell differentiation, T cell activation involved in immune response and T cell-mediated cytotoxicity in T cells (MS *vs* NC). **g** The signature enrichment score (U score) of representative pathways including leukocyte proliferation, T cell differentiation, T cell activation involved in immune response and T cell mediated cytotoxicity in T cells (MS *vs* NC). **h** CD4 and CD8 immunofluorescence staining analysis of tumor tissue sections. Quantification of the CD4 staining (**i**) and CD8 staining (j) in (**h**) (mean ± SEM, *n* = 5). **k** GO pathway enrichment analysis of DEGs in B cells showing the top 10 pathways (adjusted *p* < 0.05). **l** GSEA plot showing representative pathways including B cell proliferation, B cell activation, and B cell-mediated immunity in B cells (MS *vs* NC). **m** Bubble plot displaying the top 10 significantly downregulated pathways (MS *vs* NC) in GSEA analysis of NK cells. **n** GSEA plot showing representative pathways including NK cell-mediated immunity, NK cell activation, and NK cell-mediated cytotoxicity in NK cells (MS *vs* NC).

Herein, our scRNA-seq analysis of the lymphocytes identified seven distinct T cell subpopulations, including Gzmb-high CTLs (*Gzmb*, *Cd7*, *Xcl1*, *Fcer1g*, *Cd3g*, etc., as marker genes), Ccl5-high CTLs (*Ccl5*, *Fasl*, *Cd8a*, *Ctla2a*, *Cd8b1*, etc., as marker genes), CD8^+^ naïve T cells (*Cd8b1*, *Ms4a4b*, *Sell*, *Tpt1*, *Eef1b2*, etc., as marker genes), CD4^+^ naïve T cells (*Cd4*, *Lef1*, *Klf2*, *Tcf7*, *Satb1*, etc., as marker genes), regulatory T cells (*Tnfrsf4*, *Ctla4*, *Foxp3*, *Il2ra*, *Dusp1*, etc., as marker genes), γδT cells (*Tmem176a*, *Tmem176b*, *Tcrg-C1*, *Trdc*, *Il7r*, etc., as marker genes) and Cycling T cells (*Hist1h2ae*, *Mki67*, *Top2a*, *Hist1h2ap*, *Stmn1*, etc., as marker genes). Additionally, two B cell subgroups were identified as naïve B cells (*Cd74*, *H2-Ab1*, *Cd79a*, *H2-Aa*, *Ebf1*, etc., as marker genes) and plasma B cells (*Jchain*, *Iglc2*, *Iglc1*, *Igha*, *Igkc*, etc., as marker genes), along with a population of NK cells (*Tyrobp*, *Ncr1*, *Klrk1*, *Gzma*, *Klrd1*, etc., as marker genes) (Fig. 6a–c). As shown in Fig. 6d, the cell number of each subpopulation of T, B, and NK lymphocytes decreased in the MS tumors, compared to NC.

DEG analysis in T cells (Supplementary Table 5, Supplementary Fig. S6) and subsequent pathway analysis indicated their involvement in regulating cell proliferation, differentiation, activation, and cytotoxicity of T cells (Fig. 6e), which were further confirmed by the GSEA analysis (Fig. 6f) and the gene signature U score analysis (Fig. 6g). In addition, we applied immunofluorescence analysis of CD4 and CD8 to tumor tissue sections, demonstrating a significant suppression of both CD4^+^ and CD8^+^ T cells within the TME by stress exposure (Fig. 6h–j).

Similarly, we analyzed the B type of lymphocytes and NK cells in the TME between the MS and NC groups of tumors (Fig. 6a–c). As shown in Fig. 6d, both B cells and NK cells showed suppression by stress exposure. Trajectory analysis validated the cell differentiation direction from naïve B cells to plasma B cells (Supplementary Fig. S7a–c). DEGs analysis in B cells (Supplementary Table 6, Supplementary Fig. S7d) and subsequent pathway analysis indicated their involvement in regulating B cell-mediated immunity and immunoglobulin-mediated immune response (Fig. 6k). GSEA enrichment analysis demonstrated their involvement in regulating proliferation, activation, and immunity of B cells (Fig. 6l). GSEA enrichment analysis of DEGs in NK cells (Supplementary Table 7, Supplementary Fig. S8) indicated that these genes regulate cell killing, NK cell activation, and leukocyte-mediated cytotoxicity and immunity (Fig. 6m, n).

### Impacts of stress exposure on macrophages, neutrophils and DCs within the TME

**Fig. 7:**
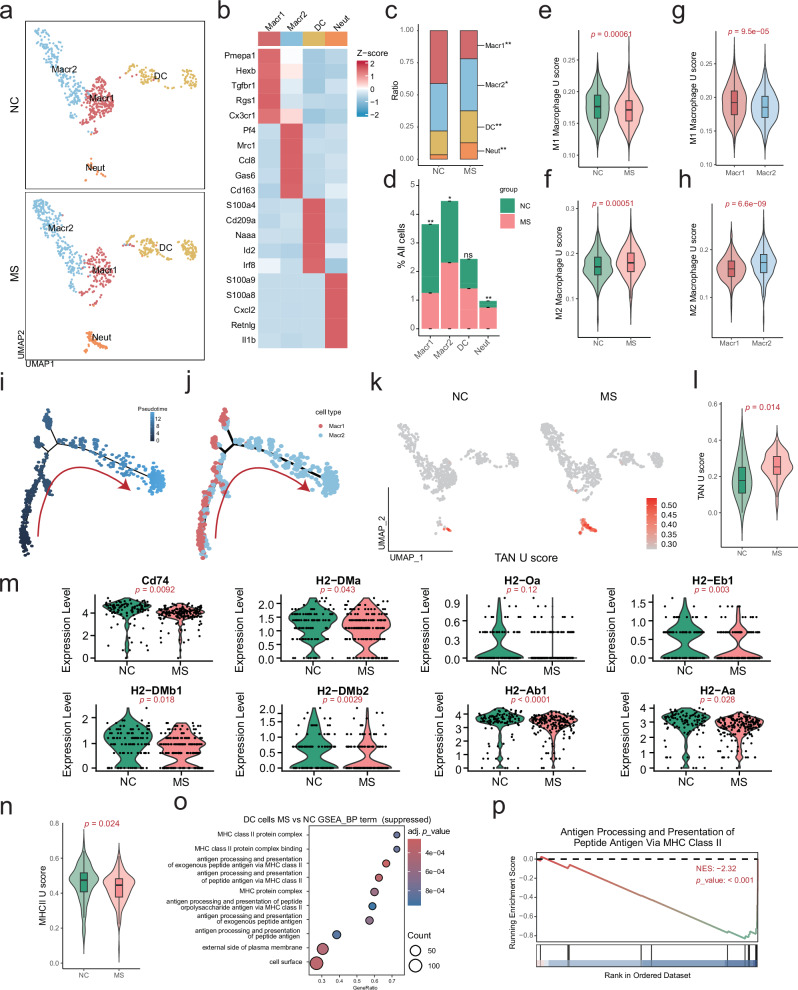
Impact of stress stimuli on myeloid cells within the TME. **a** UMAP plot of myeloid cells in the TME, grouped by cell subtypes. **b** Heatmap of the average expression levels of the marker genes used for annotation of each subtype of myeloid cells. **c** Proportion of each cell subtype in all myeloid cells. Statistical significance was indicated (Fisher’s exact test, *p < 0.05, **p < 0.01). **d** Proportion of each myeloid cell subtype in all cells. Statistical significance was indicated (Fisher’s exact test, *p < 0.05, **p < 0.01). M1 (**e**) and M2 (**f**) macrophage-associated gene set **e**nrichment U scores of all macrophages in the TME of NC and MS mice. M1 (**g**) and M2 (**h**) macrophage-associated gene set enrichment U scores of Macr1 and Macr2 subtypes of macrophages in the TME. Trajectory analysis of macrophages, displayed by pseudotime (**i**) and cell subtype (**j**). Cell differentiation direction was indicated with red arrows. **k** UMAP plot showing the expression of a gene set in tumor-associated nuetrophiles (TAN) in myeloid cells of MS and NC mice. **l** TAN U score of neutrophils in the TME of MS and NC mice. **m** The gene expression levels of *Cd74*, *H2-DMa*, *H2-Oa*, *H2-Eb1*, *H2-DMb1*, *H2-DMb2*, *H2-Ab1*, and *H2-Aa* in myeloid cells of MS and NC mice. **n** MHC II U score in DCs of MS and NC mice. **o** Bubble plot displaying the top 10 significantly downregulated pathways (MS *vs* NC) in GSEA analysis of DC cells. **p** GSEA plot showing a representative pathway of antigen processing and presentation of peptide antigen via MHC class II in DCs (MS *vs* NC).

Myeloid cells include macrophages, neutrophils, and DC in the TME. We herein clustered myeloid cells into 4 subpopulations, including macrophage Macr1 (*Cd86*, *Hexb*, *Tgfbr1*, *Rgs1*, *Cx3cr1*, etc., as marker genes), macrophage Macr2 (*Cd163*, *Mrc1*, *Ccl8*, *Gas6*, *Pltp*, etc., as marker genes), DCs (*S100a4*, *Cd209a*, *Zbtb46*, *Cd80*, *Itgax*, etc., as marker genes), and neutrophils (*S100a8*, *S100a9*, *Cxcl2*, *Retnlg*, *Il1b*, etc., as marker genes) (Fig. 7a, b, Supplementary Fig. S9a–c). Compared to the NC tumors, the MS tumors showed an increased amount of Macr2, DC, and neutrophils, while a decrease of Macr1 (Fig. 7c, d).

Gene signature U score analysis of macrophages showed a significant reduction of the M1 activity but induction of the M2 activity upon stress exposure (Fig. 7e, f). Same analysis to the Macr1 and Macr2 subpopulations indicated a stronger M1 activity in Macr1, and stronger M2 activity in Macr2 (Fig. 7g, h), suggesting the MS-induced Macr2 subpopulation having more M2 characteristics and functional activity, which was further validated by UMAP analysis of the M2 macrophage marker Cd163 (Supplementary Fig. S9a). Macrophage transition trajectory analysis indicated a transition from Macr1 to Macr2 upon stress exposure (Fig. 7i, j).

Neutrophils within the TME showed a significant induction by stress exposure (Fig. 7c, d). We compared the stress-upregulated DEGs in neutrophils (Supplementary Table 8, Supplementary Fig. S10a) with the gene signature in tumor-associated neutrophils (TANs), indicating an induction of TAN-like neutrophils within the TME by stress exposure (Supplementary Fig. S10b), which was further validated by the UMAP analysis of TAN signature U score analysis (Fig. 7k, l).

DCs are essential for antigen presentation and T-cell activation within the TME [26]. As shown in Fig. 7d, the MS tumors had more DCs within the TME than NC. Analysis of the DEGs in DC cells (Supplementary Table 9, Supplementary Fig. S11) indicated a significant suppression of a subset of MHC II complex-related genes by stress exposure, including *Cd74*, *H2-DMa*, *H2-Oa*, *H2-Eb1*, *H2-DMb1*, *H2-DMb2*, *H2-Ab1*, and *H2-Aa* (Fig. 7m). The stress-suppressed MHC II complex in DCs was further confirmed by a gene signature U score analysis (Fig. 7n) and an additional GSEA analysis (Fig. 7o, p).

## Discussion

The TME is a highly dynamic and intricate ecosystem, consisting of diverse cell types and molecular interactions that significantly influence tumor development, growth, metastasis, and responses to treatment. The impact of MS on the TME includes interactions between the neuroendocrine system, immune system, and tumor development and progression. Our previous study has demonstrated the MS-induced mammary gland tumor growth in a mouse model of breast cancer cell transplantation, in which we observed significant changes in the number and characteristics of immune cells in both peripheral blood and the TME of the stress-stimulated mice [27]. The current study applied MS stimuli to a *MMTV-PyMT* transgenic mouse model of breast cancer to investigate the effects of stress exposure on mammary tumor cells and the TME during spontaneous tumorigenesis and tumor growth in vivo.

We performed single-cell RNA sequencing on mammary tumors with or without exposure to stress stimuli. Ten major cell types including epithelial cells, endothelial cells, fibroblasts, pericytes, lymphocytes (T cells, B cells, and NK cells), and myeloid cells (macrophages, DCs, and neutrophils) were annotated from each tumor sample for further analyses of intercellular interactions, cell differentiation trajectories, differentially expressed genes (DEGs), and enriched pathways between the groups of MS and NC.

Our analysis of epithelial tumor cells not only demonstrated a stress-induced acceleration of the cell cycle but also suggested a stress-induced dedifferentiation of luminal subtype tumor cells to a subtype of cancer stem cell-like basal tumor cells with more aggressiveness. This is consistent with the literature showing that MS enhances cancer stem cell traits and cancer cell proliferation [28]. DEGs analysis of epithelial tumor cells showed a significant upregulation of those cell proliferation-regulating genes, such as *Lgals7*, *Hmgb2*, *Apoe*, *Fabp5*, and *Eif3j1* by stress exposure. GSEA analysis further highlighted the stress-enhanced activity in pathways regulating epithelial cell proliferation, cell cycle, and Wnt signaling.

Our analysis of endothelial cells and pericytes demonstrated an induction of the cell cycle and angiogenesis by stress stimulation. Endothelial cells were classified into four subtypes including Endo1, Endo2, Endo3, and Endo4 in our cases. The amount of Endo1 and Endo2 subpopulations within the TME increased upon stress exposure. The Endo1 subpopulation was proposed to be responsible for cell proliferation, and the Endo2 subpopulation for lipid metabolism and adipogenesis. In addition, our study identified Pericyte1, a novel subpopulation of pericytes, was induced by MS within the TME. Pericyte1 showed enrichment with the pathways regulating the cell cycle, EMT, and angiogenesis. Taken together, we conclude that increased cell proliferation and energy metabolism in endothelial cells and pericytes contribute to the stress-induced tumor growth.

Myeloid cells include macrophages, neutrophils, and DCs, playing essential roles in the TME. In our cases, we classified macrophages into subpopulations of Macr1 and Macr2, in which Macr1 showed more characteristics of M1 macrophages, while Macr2 seemed more like M2 macrophages. Notably, we found that stress exposure induced a transition from Macr1 to Macr2, supporting the stress-suppressed immune surveillance within the TME [29]. In addition, our analysis not only revealed an increase in the number of neutrophils but also indicated a tumor-associated neutrophil (TAN)-like phenotype in these cells. This is supported by a recent publication showing that chronic stress increases lung metastasis from disseminated cancer cells [30]. Chronic stress significantly increased neutrophil infiltration and increased neutrophil extracellular trap formation within the microenvironment, thereby establishing a metastasis-promoting TME [30]. These results suggest that MS may directly influence the TME by promoting an inflammatory response and inducing tumor-associated neutrophils, thereby accelerating tumor cell proliferation and distant metastasis.

Lymphocytes can suppress tumor development through immune surveillance, or facilitate tumor escape under immune suppression. Literature has demonstrated an attenuation of the anti-tumor immune function of T, B, and NK cells by MS [31, 32]. In consistence with the literature, we here observed a general decrease in the number of various types of lymphocytes within the TME (Fig. 6d), including T, B, and NK cells, in the mice exposed to MS. Notably, stress suppressed both naïve T cells and CTLs, indicating the stress-induced TME is not suitable for T cell expansion and survival, which may explain the T cell exhaustion and immune evasion of tumor cells in the MS group of mice. Additional analysis revealed a significant reduction in both naïve and plasma B cells within the TME of MS mice.

Our analysis of the DCs showed that stress exposure increased the number of DCs in the TME (Fig. 7d). As documented in the literature [33–36], DC functionality is highly context-dependent. Type 1 conventional DCs (cDC1s) promote antitumor immunity through cross-presentation to CD8^ +^ T cells, type 2 conventional DCs (cDC2s) activate CD4 ^+^ T cells, and plasmacytoid DCs (pDCs) modulate immune responses via type I interferon secretion. In contrast, TIDCs and monocyte-derived DCs often exhibit an immunosuppressive phenotype, characterized by downregulation of MHC-II and CD80/CD86, as well as upregulation of PD-L1, IDO, and IL-10, thereby promoting T cell tolerance. Consistent with these findings, our results demonstrated that stress exposure increased the number of DCs and decreased MHC-II-related gene expression, suggesting a functional shift toward a MoDC/TIDC-like immunosuppressive state.

Taken together, these findings further validated that MS diminishes the anti-tumor immune response by reducing lymphocyte populations and impairing their immune protection functions, thereby facilitating tumor development and tumor progression.

## Methods

### Mice

The *FVB/N-MMTV-PyMT* transgenic mice were generously provided by Professor Liu S. at Fudan University. Virgin female mice (4 weeks old, *n* = 5 per group) were randomly assigned to mental stress (MS) or negative control (NC) groups using a computer-generated randomization sequence (Microsoft Excel RAND function), with researchers blinded to group allocation during assignment. No animals or tumors were excluded from analysis; all collected data from the 40 tumors per group (8 tumors/mouse) were included without pre-established exclusion criteria. The multifocal tumor nature of this model (yielding 40 analyzable tumors/group) provided >99% power (α = 0.05) to detect the observed 51.3% increase in tumor weight (0.3639 *vs* 0.2404 g, *p* = 0.009), with variance homogeneity confirmed by F-test (*p* = 0.491). All procedures were approved by Tongji University’s Animal Care Committee ([2022] DONG YAN YU SHEN NO.046).

### Mental stress exposure

MS exposure was administered to mice in the MS group for a continuous 2 weeks. The stress stimuli included noise stimulation (80 dB for 120 min), restraint stimulation (for 120 min), elevated platform stimulation (10 cm diameter, 1.2 m height of a platform for 120 min), shaking stimulation (60 rpm for 120 min), and somatosensory stimulation (tail-clamping for 1 min, repeating 3 times within 10 min). Each mouse received one of these six stimuli unpredictably through random selection on a daily basis.

### Mouse behavior test

All behavioral tests (OFT, TST, and SPT) were conducted and analyzed by investigators blinded to group allocation. The experimenters performing the tests were different from those who handled the stress interventions, and all video recordings were coded to conceal group identity during analysis. Behavioral procedures followed established protocols [37, 38], with activity recorded using a video tracking system (SuperMaze V2.0, Shanghai, China). Automated scoring algorithms were used where possible to minimize subjective bias, and manual scoring was performed by two independent blinded researchers with inter-rater reliability >90%.

### Preparation of single cell suspension

Tumor tissues were minced and then digested at 37 °C for 40 min using an enzyme cocktail containing 150 U/mL of Collagenase IV, 20 U/mL of Dispase and 0.33 U/mL of DNase I, followed by filtering through a 40 μm strainer to obtain the single cell suspension. Acridine orange (AO) and propidium iodide (PI) were used for fluorescence staining of viable cells. Cell sorting was performed using the WOLF® Cell Sorter (NanoCellect).

### Single-cell sequencing (scRNA-seq)

Sorted single cells were diluted in DMEM/F12 with 10% FBS, and loaded on a Chromium Controller to generate single-cell gel bead emulsions, targeting 20,000–30,000 cells for each tumor tissue (*n* = 2 in the current study). Single-cell 3′ RNA-seq libraries were generated according to the manufacturer’s instructions (Chromium Single Cell 3′ Reagent v2 Chemistry Kit, 10× Genomics, Inc.), followed by a sequencing analysis to reach an average depth of ~200,000 reads per cell using Illumina Novaseq 6000 system.

### Pre-processing and quality control of scRNA-seq data

Raw reads were quality-controlled, demultiplexed, and aligned to the mm10 mouse reference genome and *PyMT* using Cellranger (version 5.0.0, 10× Genomics). The *PyMT* gene sequence was retrieved from GeneBank database (accession number AAB59900.1). Cells with less than 250 genes, or greater than 4000 genes, or over 10% reads aligning to mitochondrial genes, or gene number greater than 0.8 per UMI were filtered. DoubletFinder was employed to identify and remove doublets [39]. SCTransform function in Seurat was applied to identify genes with high variance for normalization, in which mitochondrial mapping percentage was used as a parameter for covariant correction [40]. IntegrateData function in Seurat, jointly integrating Canonical correlation analysis and Mutual nearest neighbors algorithms, was performed to correct for batch effects between samples and then merge them together [41].

### Dimensionality reduction and cluster analysis

The integrated normalized data metrics were applied for the standard analysis as described in the Seurat R package. Principal component analysis (PCA) was applied to reduce the dimensionality of the normalized scRNA-seq matrix including 3000 highly variable genes. FindClusters function in Seurat was further performed to identify cell clusters with Louvain algorithm. Based on the top 30 principal components, UMAP, a non-linear dimension reduction algorithm, was used for clustering visualization.

### Cell type annotation

The automated method SingleR (version 1.4.1) and a manual approach were both used to create annotation maps [42]. The cell-specific markers for manual annotation include: *Epcam*, *Chil1*, *Krt8* and *Krt18* for epithelial cells; *Pecam1*, *Cldn5*, *Eng*, *Cdh5* and *Emcn* for Endothelial cells; *Col1a1*, *Col1a2*, *Col3a1*, *Fn1* and *Dcn* for fibroblasts; *Acta2*, *Myl9*, *Rgs5*, *Higd1b* and *Ndufa4l2* for pericytes; *Trac*, *Trbc2*, *Cd4*, *Cd8a* and *Cd8b1* for T cells; *Klrk1*, *Klrb1c* and *Klre1* for NK cells; *Cd79a*, *Cd79b*, *Igkc* and *Ms4a1* for B cells; *Aope*, *C1qa*, *C1qb* and *C1qc* for macrophages; *Cd74*, *H2-Aa* and *H2-Ab1* for DCs; *S100a8*, *S100a9*, *Cxcl2* and *Il1b* for neutrophils; and *PyMT* for tumor cells.

### Analysis of differentially expressed genes

The FindMarkers function in Seurat was used to explore the DEGs in each cell type between the MS and NC groups. The results were visualized as volcano plots.

### Pathway enrichment analysis of DEGs

DEGs with adjust *p*-value < 0.001 and avg-diff >0.25 were used for pathway enrichment analysis. The compareCluster function in clusterProfiler was used for GO and KEGG pathway enrichment analysis. Gene set enrichment analysis (GSEA) (clusterProfiler, version 4.0.5) was used to perform gene set enrichment analyses.

### Cell-to-cell communication

CellChat (version 1.1.0) was used to analyze the cell-to-cell interactions based on receptor-ligand signaling database [43]. All cell types were explored for cell-cell interaction networks. Data of MS and NC groups were applied to CellChat respectively, and interaction strength (probability) was compared. Interactions with a *p*-value < 0.05 were considered statistically significant.

### Pseudo-time analysis

The R package Monocle (version 2.20.0) was used to construct differentiation trajectories [44]. Count data were used as input, and the differentially expressed genes were identified for dimensionality reduction using the DDRTree method. Cell trajectories were visualized and colored by pseudo-time or cell type. A pseudo-time heatmap was generated to display the pseudo-time of the marker genes in each cluster.

### Signature enrichment scoring

AddModuleScore-UCell from the UCell package was used to calculate module or signature enrichment scores at the single-cell level using the Mann-Whitney U statistic. Hallmark, Gene Ontology gene sets and KEGG pathways were obtained from the Molecular Signatures Database (MSigDB). The Cytotoxic gene sets, M1 gene sets and M2 gene sets were downloaded from Enrichr (maayanlab. cloud) [45].

### Immunofluorescence staining

Mice were perfused via the right ventricle with 50 mL of pre-cooled 1 × PBS to flush out immune cells and red blood cells from the vasculature. Mammary gland tumor tissues were then harvested, fixed in 4% paraformaldehyde (Beyotime, China), and embedded in paraffin. Tissue sections were incubated with primary antibodies at 4 °C overnight, including Ki67 (ab15580, Abcam), CD31 (ab182981, Abcam), CD4 (ab183685, Abcam), and CD8 (ab217344, Abcam). After washing with 1 × PBS, the sections were incubated with the corresponding secondary antibodies for 60 min at room temperature. Triple immunofluorescence staining was performed following the manufacturer’s protocol (AFIHC024, China). Images were captured using a Leica DM300 microscope, and quantitative analysis was conducted with Leica Application Suite X software (version 3.0.0.15697).

### Statistical analysis

Data are presented as mean ± SEM unless otherwise stated. Two-tailed *t-*test was used to analyze the independent samples between groups. For data that did not meet normal distribution requirements, such as proportions of cell subpopulations, Fisher’s exact test was employed. All the data met the respective distribution requirements of the tests applied. *p* < 0.05 was considered statistical significance.

## Supplementary information

Supplementary figures and tables legends

Supplementary Figure S1

Supplementary Figure S2

Supplementary Figure S3

Supplementary Figure S4

Supplementary Figure S5

Supplementary Figure S6

Supplementary Figure S7

Supplementary Figure S8

Supplementary Figure S9

Supplementary Figure S10

Supplementary Figure S11

Supplementary Table1. DEGs in epithelial cells (MS *vs* NC)

Supplementary Table2. DEGs in endothelial cells (MS *vs* NC)

Supplementary Table3. DEGs in fibroblasts (MS *vs* NC)

Supplementary Table4. DEGs in pericytes (MS *vs* NC)

Supplementary Table5. DEGs in T cells (MS *vs* NC)

Supplementary Table6. DEGs in B cells (MS *vs* NC)

Supplementary Table7. DEGs in NK cells (MS *vs* NC)

Supplementary Table8. DEGs in neutrophils (MS *vs* NC)

Supplementary Table9. DEGs in dendritic cells (MS *vs* NC)

## Data Availability

The datasets generated for this study are available in the GEO database (https://www.ncbi.nlm.nih.gov/geo/query/acc.cgi?acc=GSE297521). All data generated or analyzed during this study are included in this published article and its supplementary information files or are available from the corresponding author upon reasonable request.

## Code availability

R and other custom scripts for analyzing data are available upon request.
