# Supplementary material for: Single-cell RNA sequencing reveals the effects of mental stress on mouse mammary tumors and the tumor microenvironment
Source: Cell Death Discov. 2025 Jul 16;11:328. doi: 10.1038/s41420-025-02619-1 (PMC12267534; doi:10.1038/s41420-025-02619-1)
Supplement: Supplementary file 1 — Supplementary figures and tables legends [file 41420_2025_2619_MOESM1_ESM.docx]

**Supplementary figure legends.**

**Supplementary Figure S1. Impact of stress stimuli on the body weight of mice.** The body weight of the mice in the MS and NC groups at week 10. ns: no significance.

**Supplementary Figure S2. Impact of stress stimuli on epithelial tumor cells. a-b.** *Mki67* expression patterns in epithelial tumor cells of MS and NC mice. **c.** Pseudotime trajectory analysis of the top 10 marker genes in three subtypes of epithelial tumor cells showed by heatmap.

**Supplementary Figure S3. Impact of stress stimuli on endothelial cells in the TME. a-b.** *Mki67* expression patterns in endothelial cells of MS and NC mice. **c.** Pseudotime trajectory analysis of the top 10 marker genes in four subtypes of endothelial cells showed by heatmap. **d-h.** Cell-cell communication networks targeting endothelial cells through VEGF (d), ANGPT (e), VISFATIN (f), TGFβ (g), and ANGPTL (h) pathways in NC versus MS groups. Line thickness represents interaction strength, with thicker lines indicating stronger intercellular communication.

**Supplementary Figure S4. Impact of stress stimuli on fibroblasts in the TME. a-b.** UMAP plot showing the expression patterns of the representative marker genes in mCAF (a) and iCAF (b).

**Supplementary Figure S5.** **Impact of stress stimuli on** **pericytes in the TME. a.** UMAP plot of pericytes in the TME, grouped by cell subtypes. **b.** Heatmap of the average expression levels of the marker genes used for annotation of pericyte subtypes. **c.** Proportion of each pericyte subtype in all pericytes. Statistical significance was indicated (Fisher's exact test, ***p* < 0.01). **d.** Proportion of pericytes at different stages of the cell cycle. Statistical significance was indicated (Fisher's exact test, ***p* < 0.01). **e-f.** *Mki67* expression patterns in pericytes of MS and NC mice. **g-h.** Trajectory analysis of pericytes, displayed by pseudotime (g) and cell subtype (h). Cell differentiation direction was indicated with red arrows. **i.** Pseudotime trajectory analysis of the marker genes in three subtypes of pericytes showed by heatmap. **j.** Dot plot of Hallmark pathway signature enrichment score in each subtype of pericytes. **k.** Volcano plot of DEGs in pericytes in the TME between MS and NC mice. **l.** GO pathway enrichment analysis of DEGs in pericytes showing the top 10 pathways (adjusted *p* < 0.05). **m.** GSEA plot showing representative pathways including endothelial cell proliferation, extracellular matrix assembly and vascular associated smooth muscle cell migration in pericytes (MS *vs* NC). **n.** The signature enrichment score (U score) of representative pathways including endothelial cell proliferation, extracellular matrix assembly and vascular associated smooth muscle cell migration in pericytes (MS *vs* NC).

**Supplementary Figure S6. Impact of stress stimuli on T cells in the TME.** Volcano plot of DEGs in T cells in the TME between MS and NC mice.

**Supplementary Figure S7. Impact of stress stimuli on B cells in the TME.** **a-b.** Trajectory analysis of B cells, displayed by pseudotime (a) and cell subtype (b). Cell differentiation direction was indicated with red arrows. **c.** Pseudotime trajectory analysis of the marker genes in two subtypes of B cells showed by heatmap. **d.** Volcano plot of DEGs in B cells in the TME between MS and NC mice.

**Supplementary Figure S8. Impact of stress stimuli on NK cells in the TME.** Volcano plot of DEGs in NK cells in the TME between MS and NC mice.

**Supplementary Figure S9. Impact of stress stimuli on Myeloid cells in the TME. a-c.** UMAP plot showing the expression patterns of representative marker genes in macrophages (a), dendritic cells (b) and neutrophils (c).

**Supplementary Figure S10. Impact of stress stimuli on Neutrophils in the TME. a.** Volcano plot of DEGs in neutrophils in the TME between MS and NC mice. **b.** The expression levels of TAN-associated genes in neutrophils in the TME between MS and NC mice.

**Supplementary Figure S11. Impact of stress stimuli on DC cells in the TME.** Volcano plot of DEGs in DC cells in the TME between MS and NC mice.

**Supplementary table legends.**

**Supplementary Table1. DEGs in epithelial cells (MS *vs* NC).**

**Supplementary Table2. DEGs in endothelial cells (MS *vs* NC).**

**Supplementary Table3. DEGs in fibroblasts (MS *vs* NC).**

**Supplementary Table4. DEGs in pericytes (MS *vs* NC).**

**Supplementary Table5. DEGs in T cells (MS *vs* NC).**

**Supplementary Table6. DEGs in B cells (MS *vs* NC).**

**Supplementary Table7. DEGs in NK cells (MS *vs* NC).**

**Supplementary Table8. DEGs in neutrophils (MS *vs* NC).**

**Supplementary Table9. DEGs in dendritic cells (MS *vs* NC).**
