# Supplementary figures and images for: Single-cell RNA sequencing reveals the effects of mental stress on mouse mammary tumors and the tumor microenvironment
Source: Cell Death Discov. 2025 Jul 16;11:328. doi: 10.1038/s41420-025-02619-1 (PMC12267534; doi:10.1038/s41420-025-02619-1)

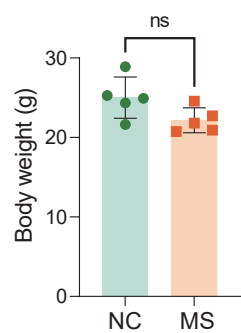

Supplement: Supplementary file 2 — Supplementary Figure S1 [file 41420_2025_2619_MOESM2_ESM.pdf]

a

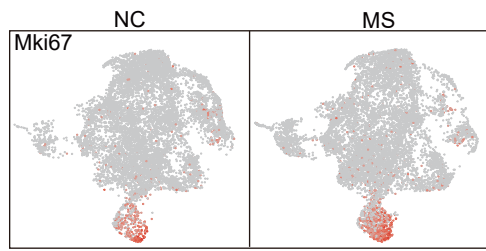

b

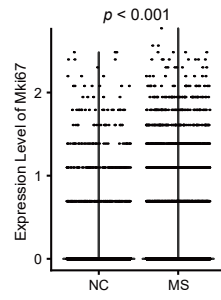

c

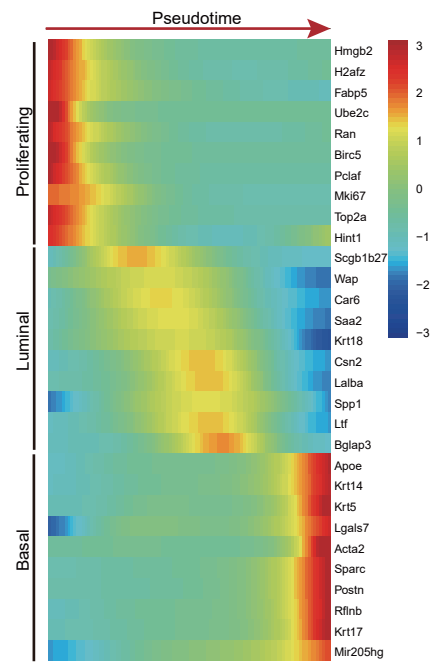

Supplement: Supplementary file 3 — Supplementary Figure S2 [file 41420_2025_2619_MOESM3_ESM.pdf]

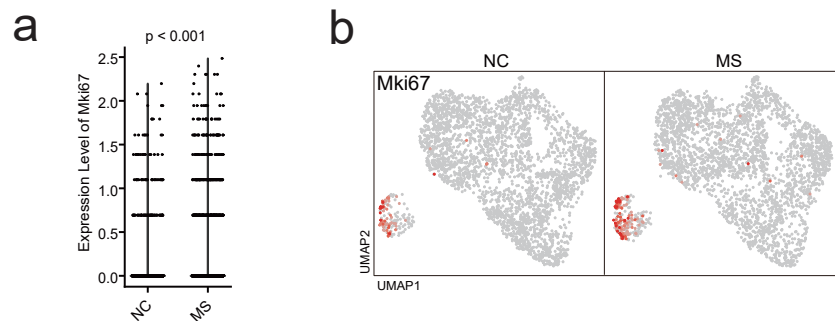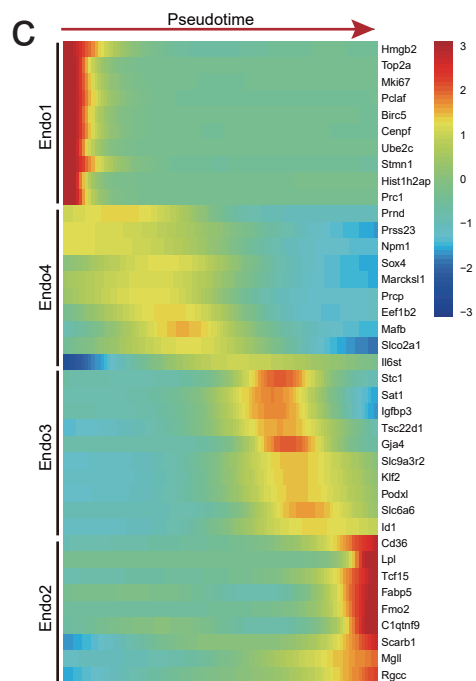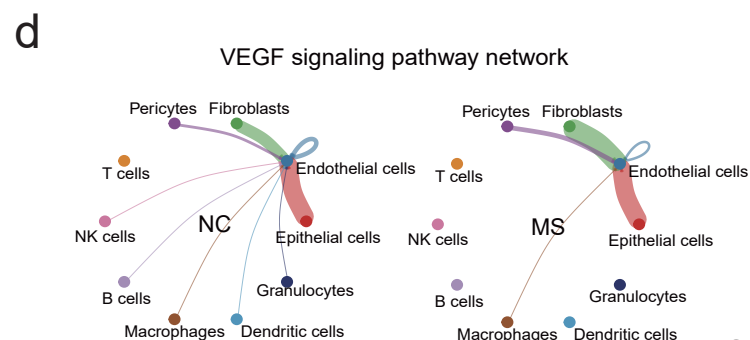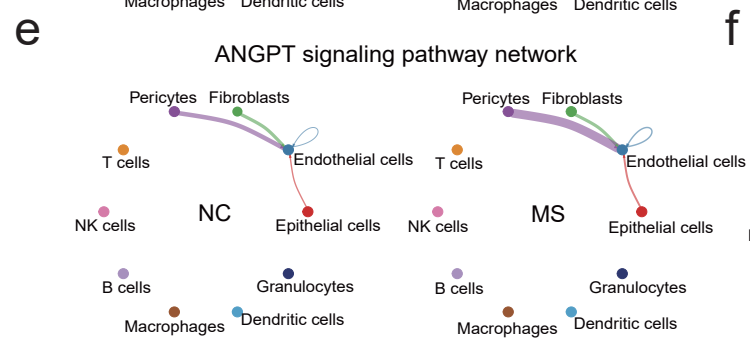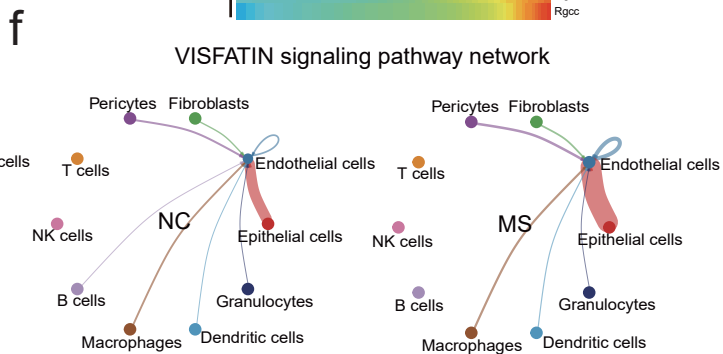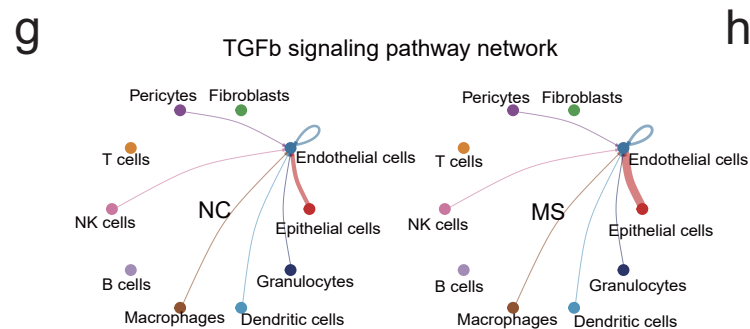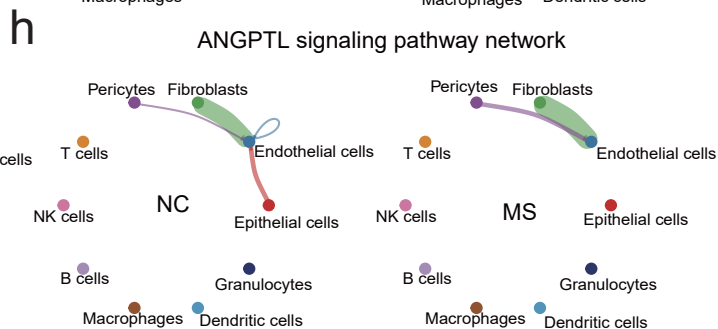

Supplement: Supplementary file 4 — Supplementary Figure S3 [file 41420_2025_2619_MOESM4_ESM.pdf]

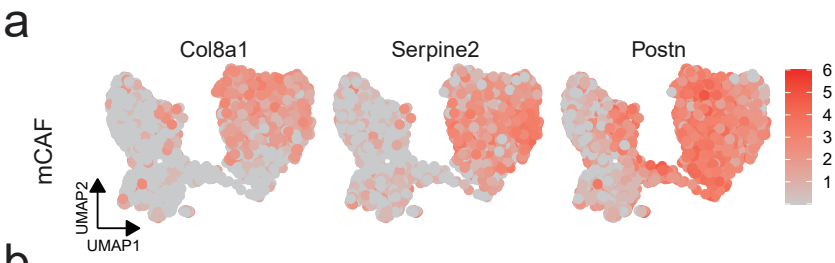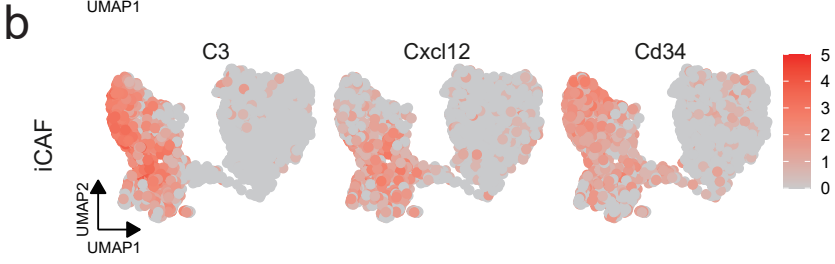

Supplement: Supplementary file 5 — Supplementary Figure S4 [file 41420_2025_2619_MOESM5_ESM.pdf]

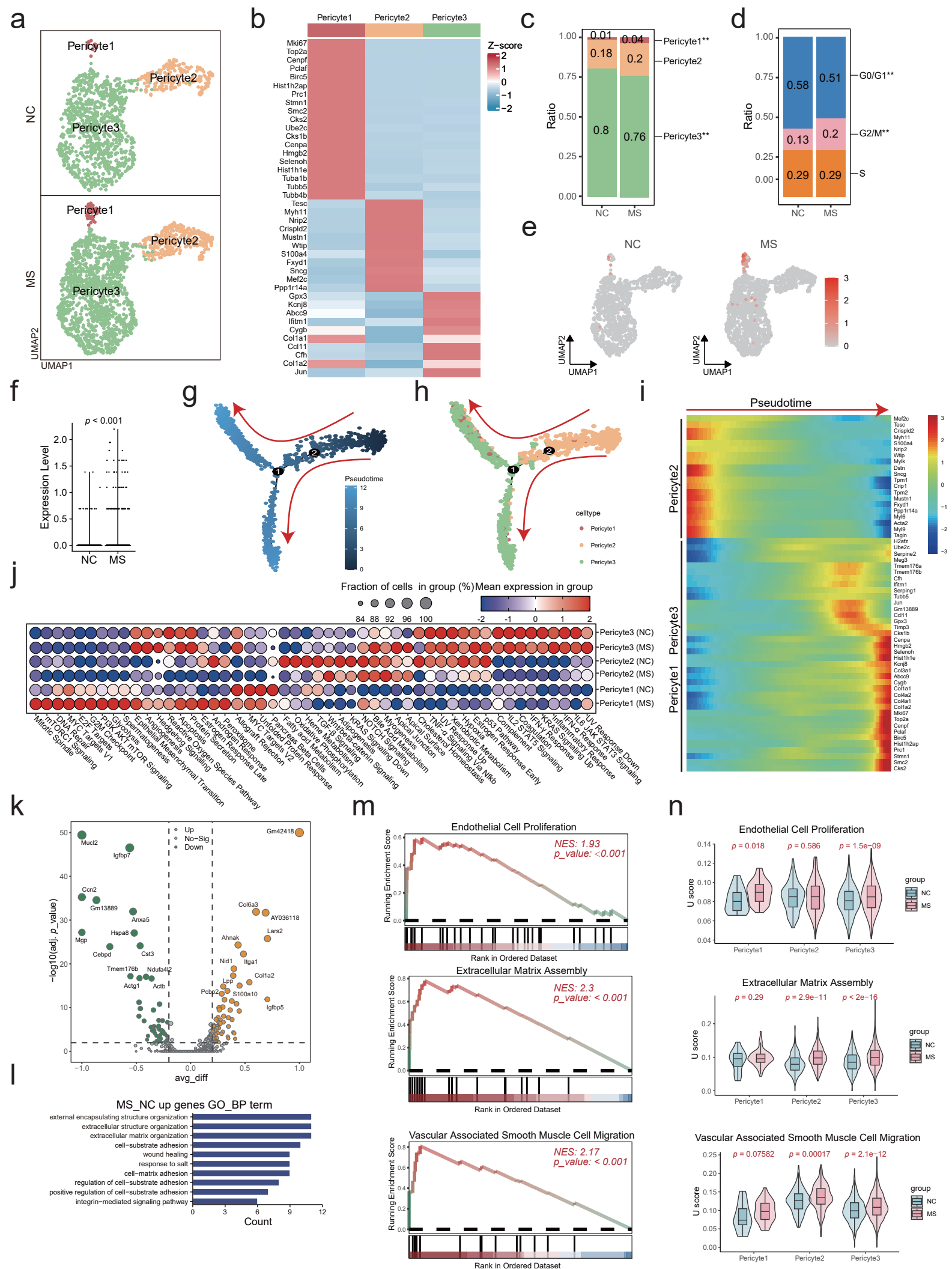

Supplement: Supplementary file 6 — Supplementary Figure S5 [file 41420_2025_2619_MOESM6_ESM.pdf]

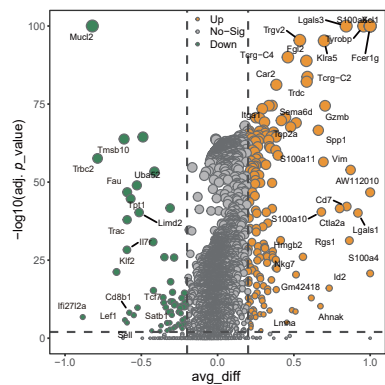

Supplement: Supplementary file 7 — Supplementary Figure S6 [file 41420_2025_2619_MOESM7_ESM.pdf]

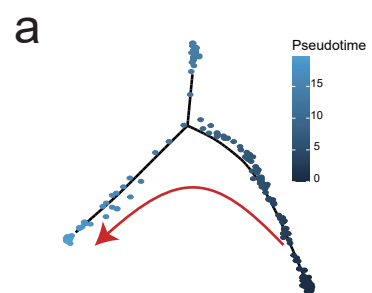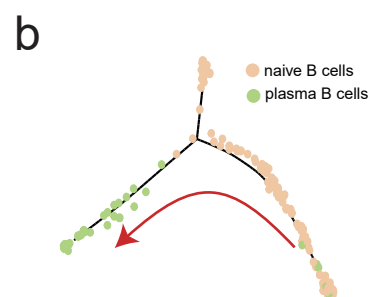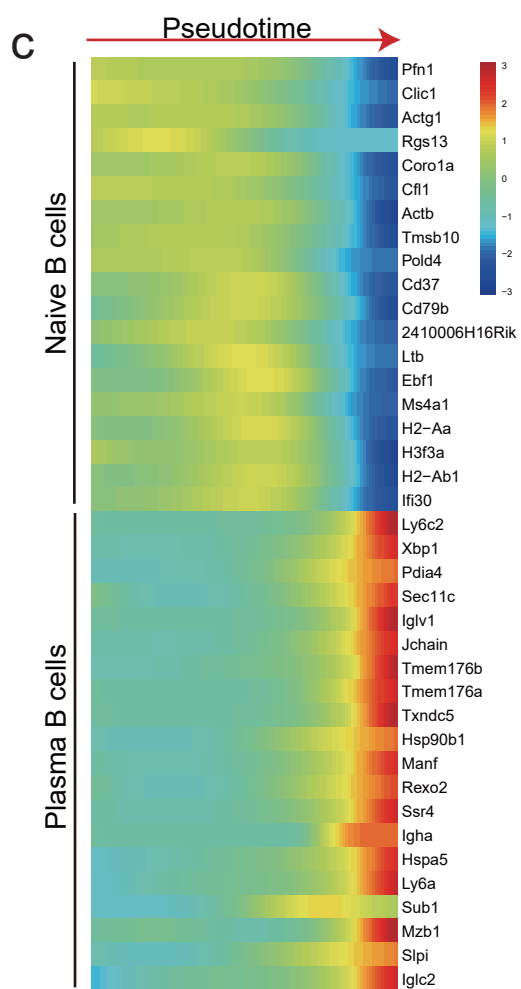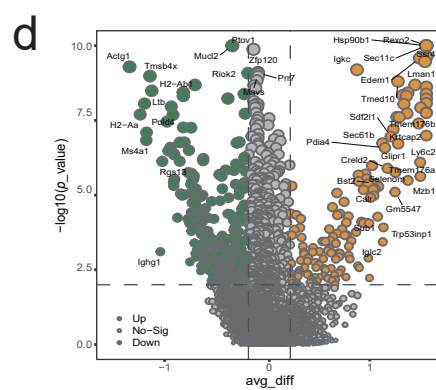

Supplement: Supplementary file 8 — Supplementary Figure S7 [file 41420_2025_2619_MOESM8_ESM.pdf]

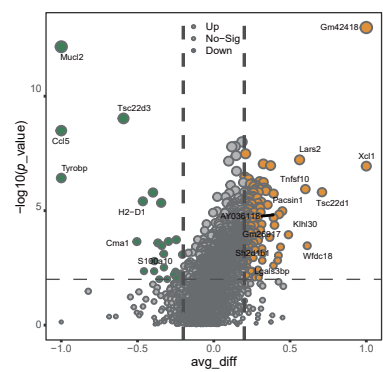

Supplement: Supplementary file 9 — Supplementary Figure S8 [file 41420_2025_2619_MOESM9_ESM.pdf]

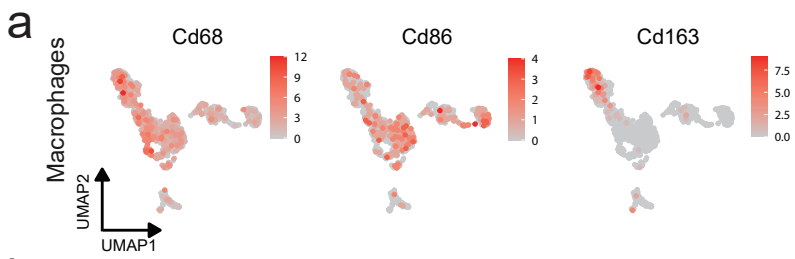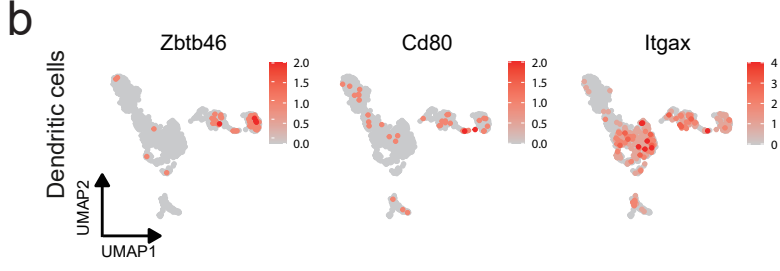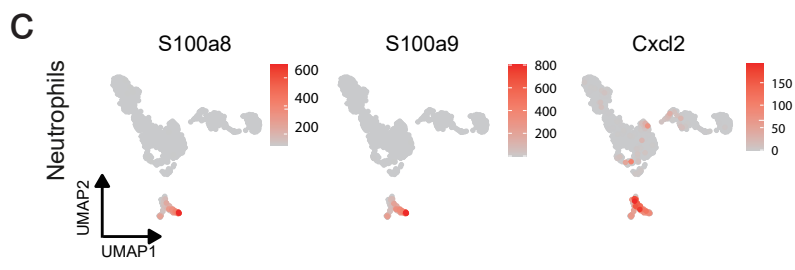

Supplement: Supplementary file 10 — Supplementary Figure S9 [file 41420_2025_2619_MOESM10_ESM.pdf]

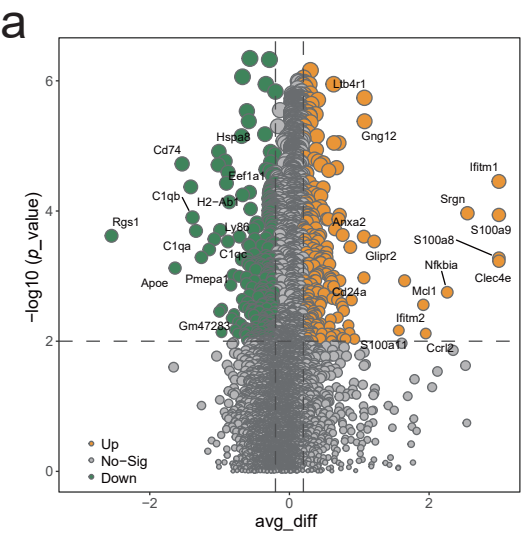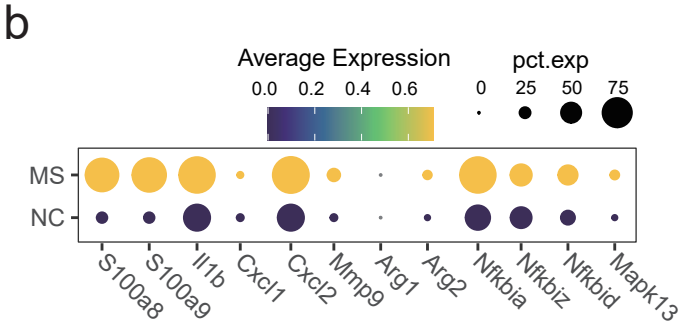

Supplement: Supplementary file 11 — Supplementary Figure S10 [file 41420_2025_2619_MOESM11_ESM.pdf]

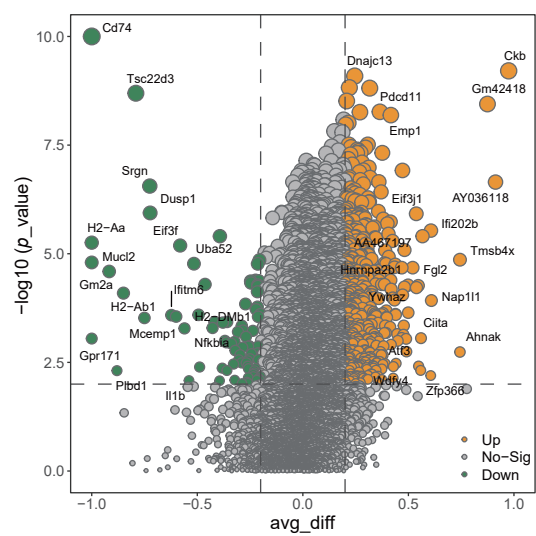

Supplement: Supplementary file 12 — Supplementary Figure S11 [file 41420_2025_2619_MOESM12_ESM.pdf]
